# Cross-Cohort Integration of Blood DNA Methylation and Sepsis Transcriptomes Prioritizes TP53INP1 as a Candidate Associated with B-Cell Transcriptomic Patterns in Pediatric HAdV-7-Associated Sepsis

**DOI:** 10.3390/pathogens15090988

**Published:** 2026-09-17

**Authors:** Peidan Hu, Bolun Huang, Wenmin Yang, Chunmin Zhang, Jinda Huang, Yiyu Yang, Feiyan Chen

**Affiliations:** Department of Pediatric Intensive Care Unit, Guangzhou Women and Children’s Medical Centre, Guangzhou Medical University, Guangzhou 510623, China

**Keywords:** HAdV-7, pediatric sepsis, DNA methylation, host–pathogen interaction, B cells, TP53INP1

## Abstract

Human adenovirus type 7 (HAdV-7) can cause severe pneumonia and sepsis in children, but the associated host epigenetic and immune alterations remain poorly defined. We profiled peripheral-blood DNA methylation in 11 children with HAdV-7-associated sepsis (6 survivors and 5 non-survivors) and 5 pediatric controls without clinically detectable HAdV-7 infection using reduced representation bisulfite sequencing. Differentially methylated genes were integrated with pediatric septic shock transcriptomic modules from GSE26440, and their cellular distribution and predicted regulatory effects were explored using the single-cell dataset GSE167363 and scTenifoldKnk. We identified 106 genes showing differential methylation across all three pairwise comparisons. Cross-cohort integration prioritized 19 candidate genes, among which TP53INP1 showed preferential expression in B cells in the external sepsis single-cell dataset. In silico perturbation of TP53INP1 was associated with changes in interferon-stimulated and antiviral genes, including TRIM22, IFI44L, and PARP14. These findings identify TP53INP1 as a hypothesis-generating candidate linking HAdV-7-associated whole-blood methylation changes with B-cell regulatory networks observed in an external sepsis dataset. Given the small discovery cohort and absence of experimental validation, the findings are exploratory and require confirmation in independent cohorts.

## 1. Introduction

Human adenoviruses (HAdVs) are non-enveloped viruses belonging to the family Adenoviridae and contain a linear double-stranded DNA genome [1]. HAdVs are important respiratory pathogens in children and can cause a broad spectrum of disease ranging from self-limited respiratory infection to severe pneumonia and critical illness. Among the circulating HAdV types, HAdV-7 has been repeatedly associated with more severe lower respiratory tract disease in pediatric populations [2,3,4]. Molecular epidemiological surveillance in Guangzhou showed a shift in the predominant HAdV subtype from HAdV-3 to HAdV-7 during 2018–2019, with HAdV-7 infection associated with greater disease severity [2]. Studies from other regions of China have similarly reported an association between HAdV-7 and severe pediatric pneumonia and respiratory failure [3,4]. However, the host molecular alterations associated with severe HAdV-7 infection and sepsis-related organ dysfunction remain incompletely understood.

The rapid clinical deterioration observed in septic patients is fundamentally driven by systemic immune dysregulation [5,6]. Current evidence indicates that tissue damage resulting from HAdV-7 infection is associated with the aberrant activation of the host’s immune system, with a particular emphasis historically placed on the innate immune response [7]. However, the pathophysiology of sepsis extends far beyond initial hyperinflammation; it is characterized by a broad immune dysfunction that involves quantitative and functional alterations in both innate and adaptive immune cells [8]. Elucidating the deeper regulatory networks that govern this broad-spectrum immune exhaustion may help inform future therapeutic strategies [9].

Epigenetic modifications act as important regulators of gene expression during disease progression, with DNA methylation representing a widely studied epigenetic modification relevant to disease-associated molecular changes [10]. Previous studies have shown that DNA methylation is associated with immune activation and immune tolerance [10,11]. Furthermore, examining methylation profiles from peripheral blood offers an accessible approach to explore the pathogenesis of systemic diseases and identify biomarkers associated with disease severity [10,11,12]. Nevertheless, to date, epigenetic profiling has rarely been applied to explore the molecular landscape of children with HAdV-7-associated sepsis.

To explore potential molecular links between HAdV-7-associated methylation alterations and sepsis-related immune dysregulation, we combined RRBS-derived methylation data from the pediatric HAdV-7 cohort with independent public bulk and single-cell transcriptomic datasets. The external datasets were used for cross-cohort candidate prioritization and cellular-context exploration rather than direct validation in the same patients. This exploratory framework was further complemented by in silico network perturbation to generate hypotheses regarding TP53INP1-associated B-cell regulatory programs.

## 2. Materials and Methods

### 2.1. Subjects

This retrospective observational study analyzed archived whole-blood samples and clinical data from pediatric patients admitted to the pediatric intensive care unit (PICU) of Guangzhou Women and Children’s Medical Centre, Guangzhou Medical University, between April 2018 and April 2020. The archived samples had been collected and stored under a broader approved research protocol on severe viral infections (Approval No. 202054301). Children aged >30 days and <18 years were included if they had severe pneumonia, laboratory-confirmed HAdV-7 infection, and infection-associated acute organ dysfunction requiring intensive care. HAdV infection was detected by PCR using throat-swab or lower-respiratory-tract specimens, and HAdV-7 typing was based on the institutional molecular diagnostic workflow described previously [2].

The term “HAdV-7-associated sepsis” was used because HAdV-7 could not be established as the sole cause of systemic organ dysfunction. The diagnosis was interpreted according to established pediatric sepsis frameworks based on severe infection accompanied by acute organ dysfunction [13,14].

Eleven children with HAdV-7-associated sepsis with available archived whole-blood samples were included in the RRBS analysis and classified as survivors (n = 6) or non-survivors (n = 5) according to in-hospital outcome. Blood samples were collected at PICU admission and stored at −80 °C. Five pediatric controls undergoing routine physical examination at the same hospital were also included. Because complete archived information on screening numbers, quantitative viral load, coinfections, and some pre-admission treatments was unavailable, these factors could not be systematically analyzed and are acknowledged as study limitations.

The broader research protocol under which the biospecimens were collected was approved by the Ethics Committee of Guangzhou Women and Children’s Medical Centre (Approval No. 202054301), with an approved study period from 1 April 2018 to 31 March 2021. The present study represents a retrospective analysis of archived biospecimens and clinical data obtained within this research framework. Written informed consent for participation and research use of biospecimens was obtained from the parents or legal guardians of all participants in accordance with the approved protocol. The study was conducted in accordance with the Declaration of Helsinki and relevant institutional guidelines.

### 2.2. RRBS Library Construction and Bioinformatics Processing

Genomic DNA was extracted from peripheral whole-blood samples using the QIAamp DNA Blood Mini Kit (Qiagen, Hilden, Germany). RRBS libraries were prepared according to established procedures [15]. Briefly, 1 μg of genomic DNA was digested with 100 U of MspI restriction enzyme (New England Biolabs, Ipswich, MA, USA) at 37 °C for 16 h. Following end repair, dATP addition, and methylated-adapter ligation, DNA fragments of 160–350 bp were size-selected from 2% agarose gels. Bisulfite conversion was performed using the EZ DNA Methylation-Gold Kit (Zymo Research, Irvine, CA, USA) according to the manufacturer’s instructions. The converted DNA was amplified using JumpStart Taq DNA Polymerase (Sigma-Aldrich, St. Louis, MO, USA) for 13 cycles (94 °C for 30 s, 58 °C for 30 s, and 72 °C for 30 s). Library quality and concentration were assessed using an Agilent 2100 Bioanalyzer (Agilent Technologies, Santa Clara, CA, USA) and quantitative PCR, respectively. The final RRBS libraries were sequenced on an Illumina HiSeq X Ten platform (Illumina, San Diego, CA, USA) using 150-bp paired-end reads.

Raw sequencing reads containing >30% ambiguous bases or low-quality bases (Q < 20) in >10% of the sequence were removed. Adapter sequences were trimmed using Cutadapt version 1.9 [16]. Clean reads were aligned to the human reference genome GRCh38 (UCSC hg38) using BSMAP version 2.73 [17], and only uniquely mapped reads containing the expected terminal MspI restriction sites were retained. CpG sites with a sequencing depth ≥ 5× were included in downstream methylation analysis. The methylation level at each CpG site was calculated as the proportion of methylated cytosine reads relative to the total number of methylated and unmethylated reads.

Differentially methylated regions (DMRs) were identified using Metilene version 0.2-7 [18], which uses recursive segmentation of intergroup methylation differences and a two-dimensional Kolmogorov–Smirnov test to assess the statistical significance of candidate regions. DMRs were required to contain at least five CpG sites, with an inter-CpG distance ≤ 300 bp, an absolute methylation difference > 0.10, and a Benjamini–Hochberg-adjusted Q-value < 0.05. Gene promoters were defined as regions extending from 2 kb upstream to 0.5 kb downstream of the transcription start site. Genes were designated as differentially methylated genes (DMGs) when at least one qualifying DMR showed >50% sequence overlap with the corresponding promoter or gene body. GO enrichment analysis of DMGs was performed using a hypergeometric test, with a Benjamini–Hochberg false discovery rate (FDR) < 0.05 considered statistically significant.

Because complete leukocyte-subset data were unavailable for the archived cohort, formal adjustment for blood-cell composition was not performed. Accordingly, differential methylation findings were interpreted as whole-blood-associated signals rather than evidence of cell-intrinsic epigenetic alterations.

### 2.3. WGCNA and Candidate Gene Prioritization

To prioritize methylation-associated candidates using an independent transcriptomic context, we performed cross-cohort analysis using the pediatric septic shock dataset GSE26440. The pediatric septic shock transcriptomic dataset GSE26440 was downloaded from the Gene Expression Omnibus (GEO) database [19,20,21]. The dataset contains whole-blood gene-expression profiles from 98 children with septic shock and 32 healthy controls generated using the Affymetrix Human Genome U133 Plus 2.0 Array platform (GPL570; Affymetrix, Santa Clara, CA, USA). The publicly available expression data had been processed using robust multi-array average (RMA) normalization, with patient samples subsequently normalized to the median values of the healthy controls. The healthy controls were used for normalization and were not included in the mortality-associated WGCNA analysis.

WGCNA was performed on the 98 septic-shock samples using WGCNA version 1.72-1 in R version 4.2.2. A signed co-expression network was constructed using Pearson correlations, with a soft-thresholding power of β = 6, a minimum module size of 30 genes, and a module-merging threshold of 0.25. In-hospital outcome was coded as 0 for survivors and 1 for non-survivors. Module eigengenes were correlated with mortality using Pearson correlation.

The turquoise module was the largest co-expression module in the original analysis and contained 5451 genes. Because its correlation with mortality was weak and non-significant, it was not interpreted as a mortality-associated module in the revised analysis. Instead, it was retained as a broad exploratory co-expression context for cross-cohort candidate prioritization. Intersection with the 106 RRBS-derived DMGs yielded 19 candidate genes for subsequent single-cell contextualization. This overlap was used to refine the candidate set across independent molecular datasets and was not interpreted as a formal enrichment test. No formal module-preservation analysis was performed, and the WGCNA-based prioritization was therefore considered exploratory.

### 2.4. Single-Cell RNA Sequencing (scRNA-seq) Data Preprocessing and Clustering

To delineate the cellular specificities of the identified candidate genes, the public scRNA-seq dataset GSE167363 was downloaded and analyzed [22]. The GSE167363 dataset comprised samples from 7 individual donors, including 2 healthy controls and 5 patients with sepsis. Because the sepsis patients were sampled longitudinally, the dataset contained multiple samples from some donors. Single-cell data were processed using Seurat version 4.3.0 in R version 4.2.2 [23]. Cells expressing 200–6000 genes and containing less than 15% mitochondrial transcripts were retained. Gene counts were log-normalized with a scale factor of 10,000, and the top 2000 highly variable genes were selected. After sample integration and data scaling, principal component analysis was performed. The first 20 principal components were used for neighbor construction, clustering at a resolution of 0.15, and uniform manifold approximation and projection visualization.

### 2.5. Cell-Type Annotation and Exploratory Cell-Level Expression Analysis

Cluster marker genes were identified using the FindAllMarkers function with the Wilcoxon rank-sum test. Marker genes were required to be expressed in at least 10% of cells, with an average log2 fold change greater than 0.25 and an adjusted *p*-value below 0.05. Cell types were annotated using canonical markers and the CellMarker 2.0 database, including CD3D and CD3E for T cells, NKG7 and GNLY for cytotoxic T/NK cells, CD79A and MS4A1 for B cells, LST1 and LYZ for monocytes, and PPBP and PF4 for platelets [24]. The expression of the 19 prioritized candidate genes was examined across the annotated cell populations. All 19 intersecting genes were carried forward without a prespecified composite ranking score. TP53INP1 was selected for focused downstream analysis because it showed a prominent B-cell-enriched expression pattern in the external single-cell dataset. This selection was exploratory and was not based on the highest WGCNA gene significance or methylation effect size. TP53INP1 expression was predominantly enriched in B-cell clusters; therefore, B cells were subsetted for further analysis. Exploratory cell-level expression differences between sepsis and healthy-control B cells were assessed using FindMarkers with the Wilcoxon rank-sum test, using an absolute average log2 fold change >0.25 and a Benjamini–Hochberg-adjusted *p* value < 0.05 for visualization and functional exploration. Because cells from the same donor are not statistically independent, these results were not interpreted as donor-level differential-expression inference. Functional enrichment analysis was performed using clusterProfiler version 4.6.2, with Benjamini–Hochberg-adjusted *p*-values below 0.05 considered significant [25].

### 2.6. Computational Network Perturbation Analysis Using scTenifoldKnk

The predicted regulatory role of TP53INP1 in sepsis-associated B cells was evaluated using scTenifoldKnk version 1.0.1 [26]. The raw count matrix of B cells from the external sepsis dataset was used as input, and genes expressed in fewer than 25 cells were excluded. Gene regulatory networks were constructed using 10 networks, with 500 cells used for each network and three principal components retained. Tensor decomposition was performed with a rank of 3, and the original and TP53INP1-perturbed networks were compared using two-dimensional manifold alignment.

Genes were ranked according to their manifold-alignment perturbation distance. The archived analysis used nominal *p*-values for exploratory visualization rather than a multiple-testing-adjusted significance threshold. Accordingly, the perturbation results were interpreted as hypothesis-generating computational predictions and were not considered evidence of experimentally validated gene regulation or biological causality. Functional enrichment of the predicted perturbed gene set was performed using clusterProfiler.

### 2.7. Statistical Analysis

Statistical analyses were performed in R version 4.2.2 unless otherwise specified. Multiple testing was controlled using the Benjamini–Hochberg procedure where applicable. WGCNA module–trait associations were assessed using Pearson correlation. Single-cell marker and exploratory cell-level expression analyses were performed using Wilcoxon rank-sum tests with Benjamini–Hochberg adjustment. scTenifoldKnk results were interpreted using nominal *p* values as exploratory outputs. Given the small size of the clinical cohort, clinical characteristics were summarized descriptively without formal between-group hypothesis testing.

## 3. Results

### 3.1. Clinical Characteristics of HAdV-7-Associated Sepsis Patients

This study included 11 children with HAdV-7-associated sepsis, including 6 survivors and 5 non-survivors. Detailed individual-level clinical characteristics and treatment information are presented in Table 1, while clinical characteristics stratified by survival status are summarized in Table 2. All patients developed ARDS and required invasive mechanical ventilation and vasoactive support. ECMO was used in 3 of 6 survivors (50.0%) and 4 of 5 non-survivors (80.0%). The median PaO_2_/FiO_2_ ratio was 93.5 (IQR, 68.3–96.3) among survivors and 66.0 (IQR, 36.0–72.0) among non-survivors. Blood samples used for RRBS were collected at PICU admission. Because of the small sample size, clinical characteristics are presented descriptively without formal between-group hypothesis testing.

### 3.2. Identification of Differentially Methylated Genes

**Figure 1:**
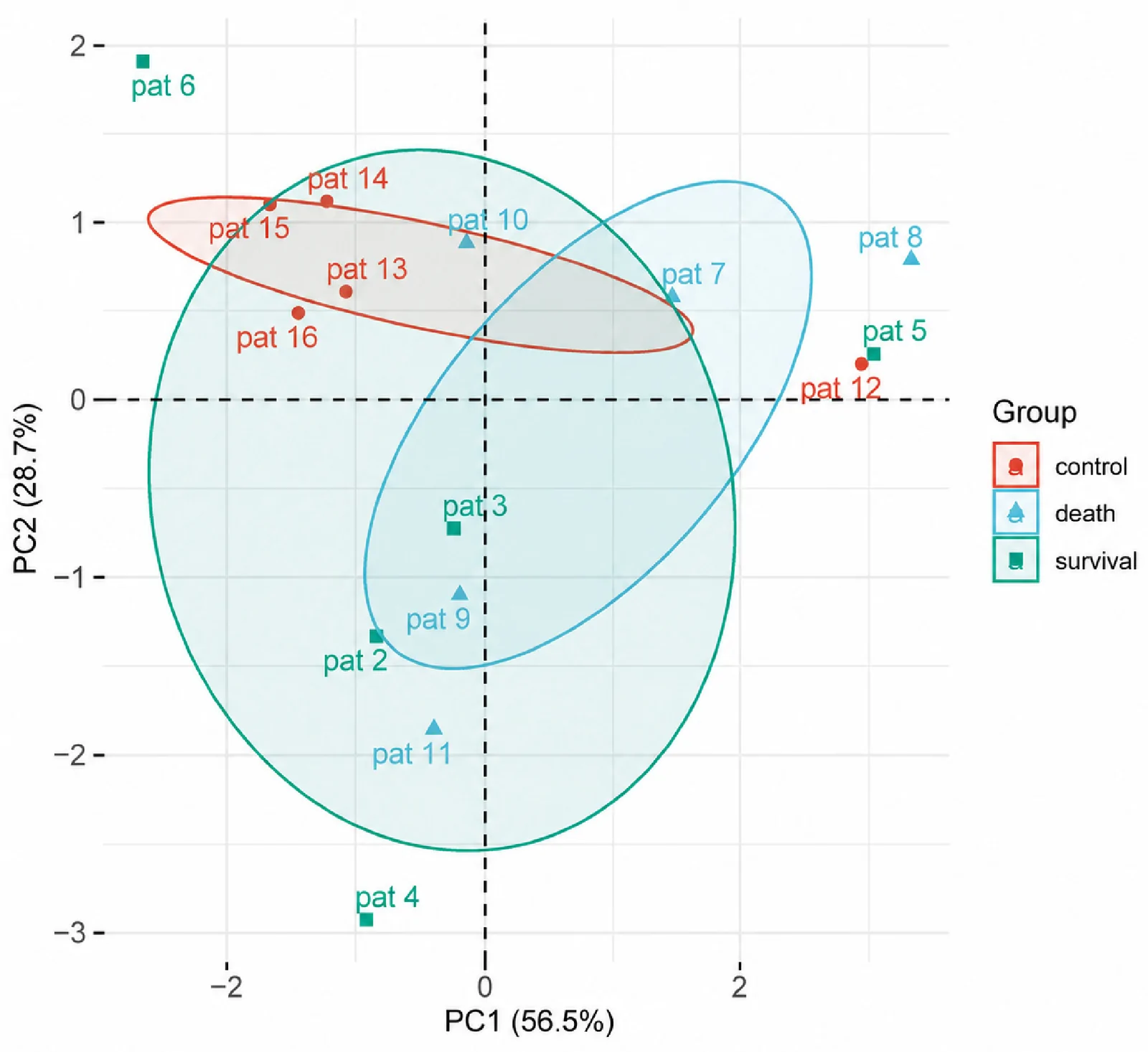
Principal component analysis (PCA) of whole-blood DNA methylation profiles from controls (n = 5), survivors (n = 6), and non-survivors (n = 5). PC1 and PC2 explained 56.5% and 28.7% of the variance, respectively. Each point represents one participant. PCA was interpreted descriptively.

**Figure 2:**
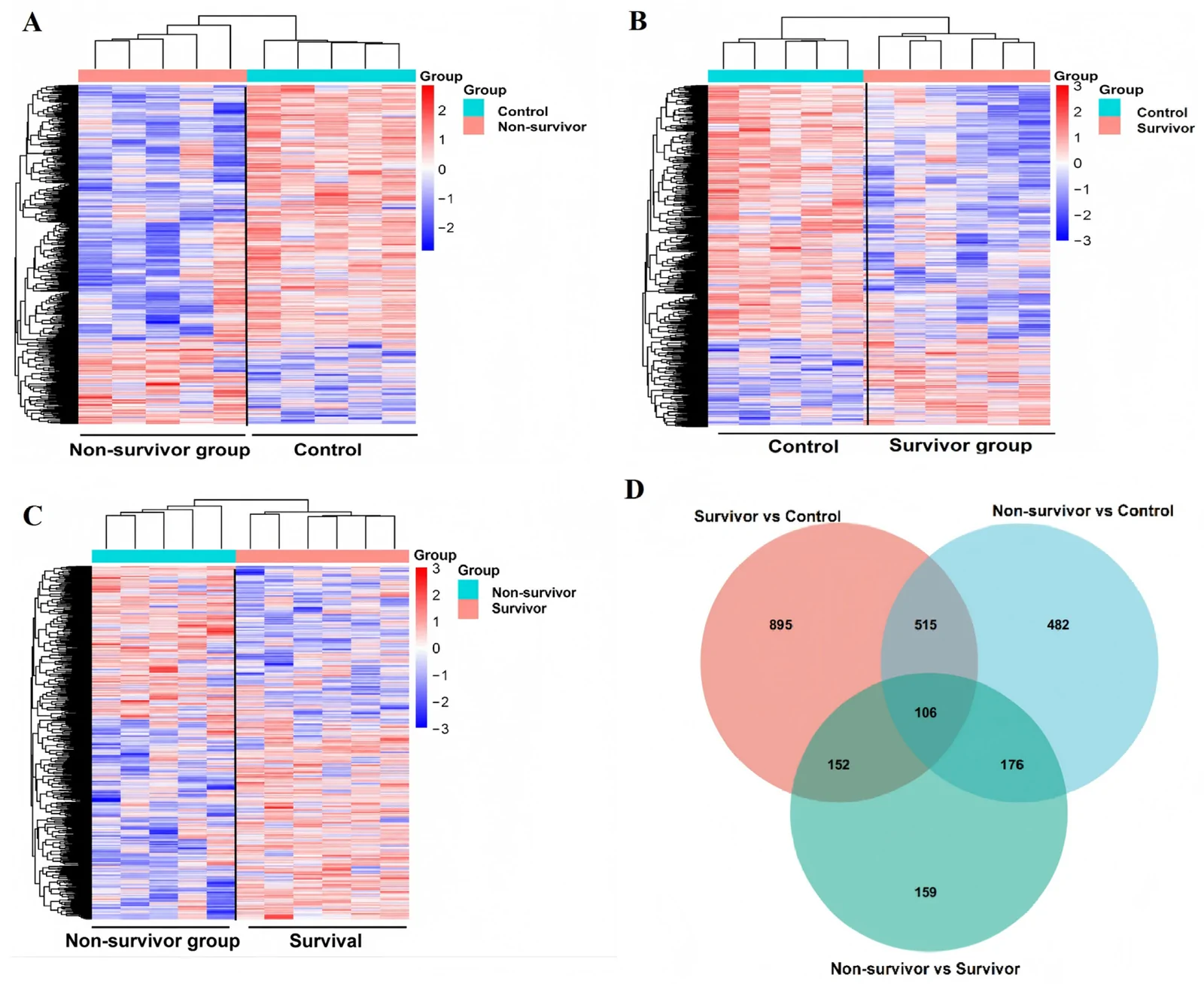
Unsupervised clustering and intersection of differential methylation signals. (**A**) Non-survivor (n = 5) vs. Control (n = 5). (**B**) Survivor (n = 6) vs. Control (n = 5). (**C**) Non-survivor (n = 5) vs. Survivor (n = 6). Red and blue indicate relatively higher and lower scaled methylation levels, respectively. (**D**) Venn diagram showing the overlap of DMGs across the three comparisons, yielding 106 shared DMGs.

To characterize whole-blood methylation differences associated with clinical outcome, RRBS methylation profiles were compared among the control, survivor, and non-survivor groups. PCA showed partial separation among the three groups, although substantial overlap remained between the survivor and non-survivor groups (Figure 1). PC1 and PC2 explained 56.5% and 20.7% of the total variance, respectively. No formal clustering-performance metric was calculated; therefore, the PCA was interpreted descriptively rather than as evidence of distinct molecular separation. Unsupervised hierarchical clustering further illustrated group-associated methylation patterns (Figure 2A–C). DMGs identified in the Survivor vs. Control, Non-survivor vs. Control, and Non-survivor vs. Survivor comparisons were intersected, yielding 106 genes shared across all three comparisons (Figure 2D). The complete list of the 106 shared DMGs is provided in Supplementary Data S1. These genes were subsequently used for cross-cohort candidate-gene prioritization. Because statistical significance across all three pairwise comparisons does not necessarily imply concordant methylation direction, the 106-gene intersection was used as a candidate-prioritization set rather than interpreted as a uniformly directional methylation signature.

### 3.3. Prioritization of Candidate Genes via WGCNA and Methylation Intersection

**Figure 3:**
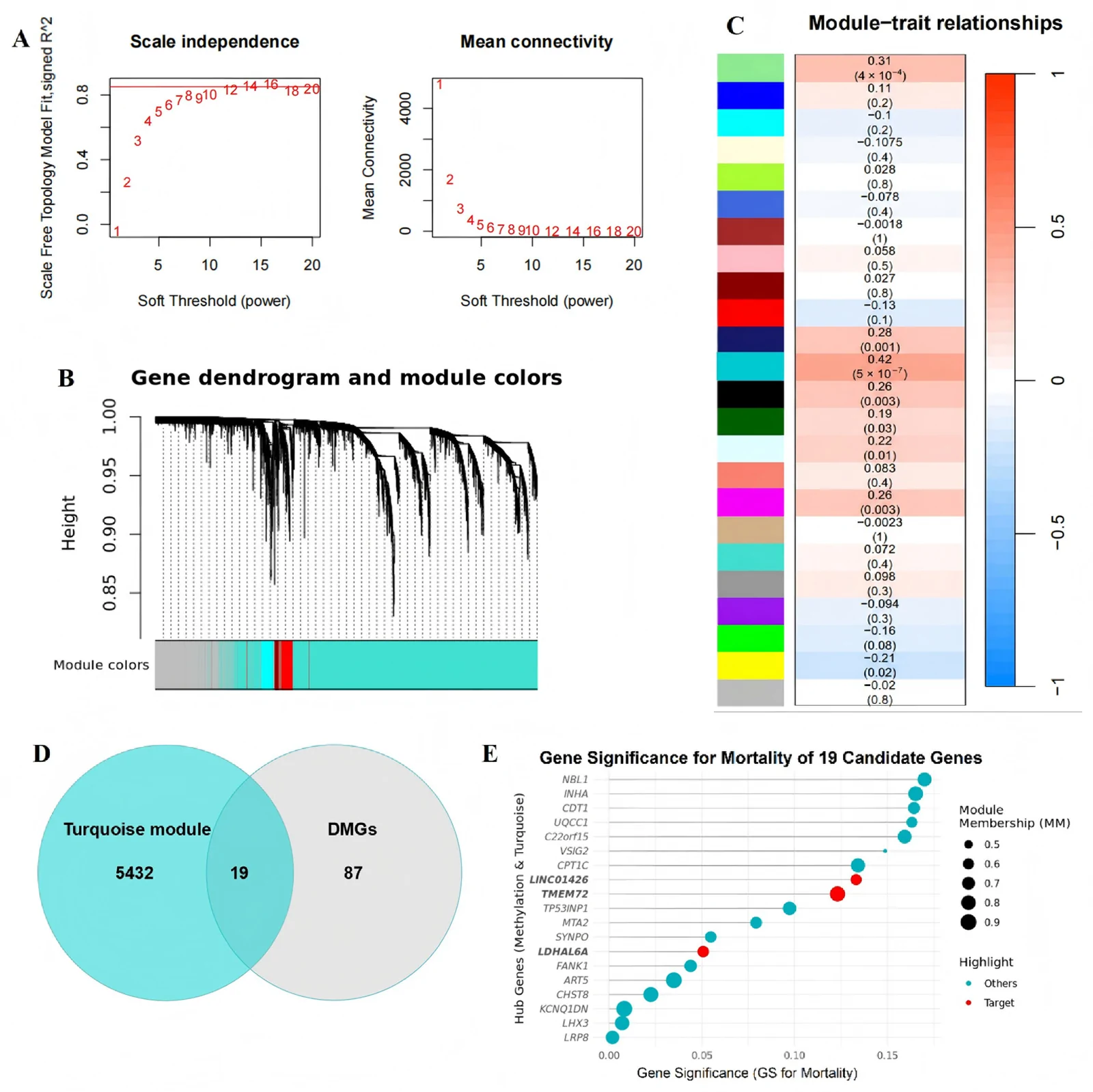
WGCNA-based co-expression analysis and candidate-gene prioritization in GSE26440. WGCNA was performed using 98 pediatric septic-shock samples. (**A**) Soft-threshold selection. (**B**) Gene dendrogram and module assignment. (**C**) Module–mortality relationships based on Pearson correlation. (**D**) Intersection of 5451 turquoise-module genes with 106 RRBS-derived DMGs, yielding 19 candidate genes. (**E**) Gene significance for mortality among the 19 candidates; point size represents module membership (MM).

WGCNA of the GSE26440 septic-shock cohort identified multiple co-expression modules (Figure 3A–C). The turquoise module was the largest module, containing 5451 genes, but showed only a weak and non-significant correlation with in-hospital mortality (r = 0.072, *p* = 0.40). Accordingly, this module was not interpreted as a mortality-associated module. Instead, it was used as an exploratory co-expression context for cross-cohort candidate prioritization. Intersection of the 5451 turquoise-module genes with the 106 RRBS-derived DMGs yielded 19 candidate genes, including TP53INP1 (Figure 3D,E). This cross-cohort overlap provided an additional layer of candidate refinement before evaluation in the external single-cell dataset. The overlap was used for exploratory prioritization rather than interpreted as a formal statistical enrichment test. All 19 genes were subsequently examined in the external single-cell dataset to assess their cell-type expression patterns. The complete list of the 19 intersecting candidate genes is provided in Supplementary Data S2. TP53INP1 was not the highest-ranked gene by WGCNA gene significance; its subsequent prioritization was based on its B-cell-enriched expression pattern in the external single-cell analysis.

### 3.4. Single-Cell Transcriptomic Characterization of the External Sepsis Dataset

**Figure 4:**
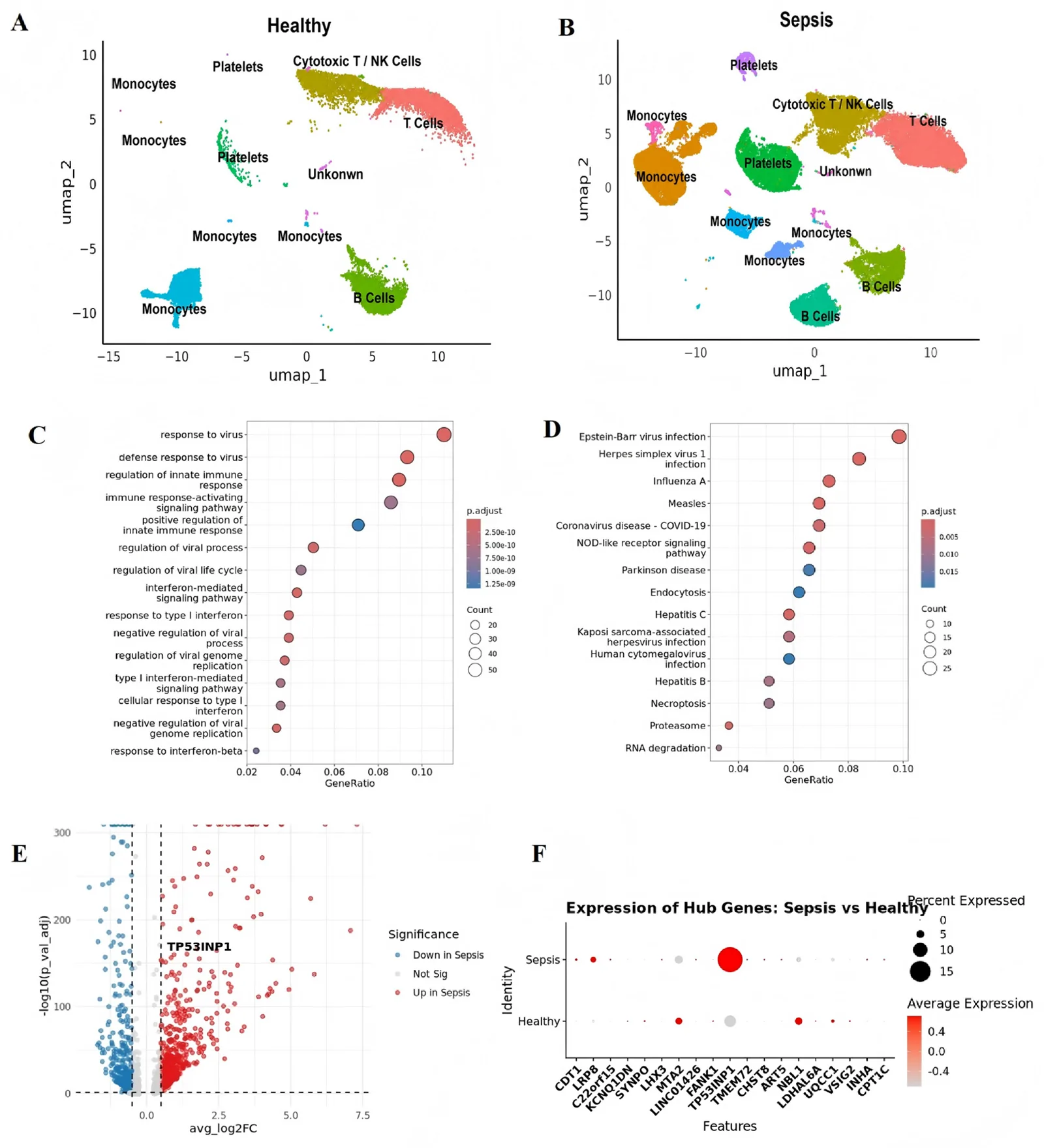
Exploratory single-cell transcriptomic analysis of GSE167363 comprising 7 donors (2 healthy controls and 5 patients with sepsis). (**A**,**B**) UMAP visualization of healthy and sepsis cells; colors indicate annotated cell populations. (**C**,**D**) GO-BP and KEGG enrichment of exploratory cell-level expression differences; point size represents gene count and color represents Benjamini–Hochberg-adjusted *p* value. (**E**) Volcano plot of exploratory cell-level expression differences identified using the Wilcoxon rank-sum test with Benjamini–Hochberg correction. (**F**) Expression patterns of the 19 candidate genes; point size represents the percentage of expressing cells and color represents average expression.

UMAP visualization was used to display the cellular landscape of PBMCs in the healthy-control and sepsis samples from the external GSE167363 dataset (Figure 4A,B). The apparent distribution of cell populations in the UMAP embeddings was considered descriptive and was not interpreted as evidence of altered cell-type composition, because donor-level cell-proportion analysis was not performed.

Exploratory cell-level expression analysis revealed genes associated with broad immune and stress-response programs. GO-BP enrichment highlighted pathways related to immune and antiviral responses (Figure 4C), whereas KEGG enrichment identified several inflammatory and immune-associated pathways (Figure 4D). The distribution of cell-level expression differences was visualized using a volcano plot (Figure 4E). The expression patterns of the 19 cross-cohort candidate genes were subsequently examined across conditions (Figure 4F). These analyses were used for cellular contextualization and hypothesis generation rather than donor-level differential-expression inference.

### 3.5. Cell-Type Annotation and B-Cell-Enriched Expression of TP53INP1

**Figure 5:**
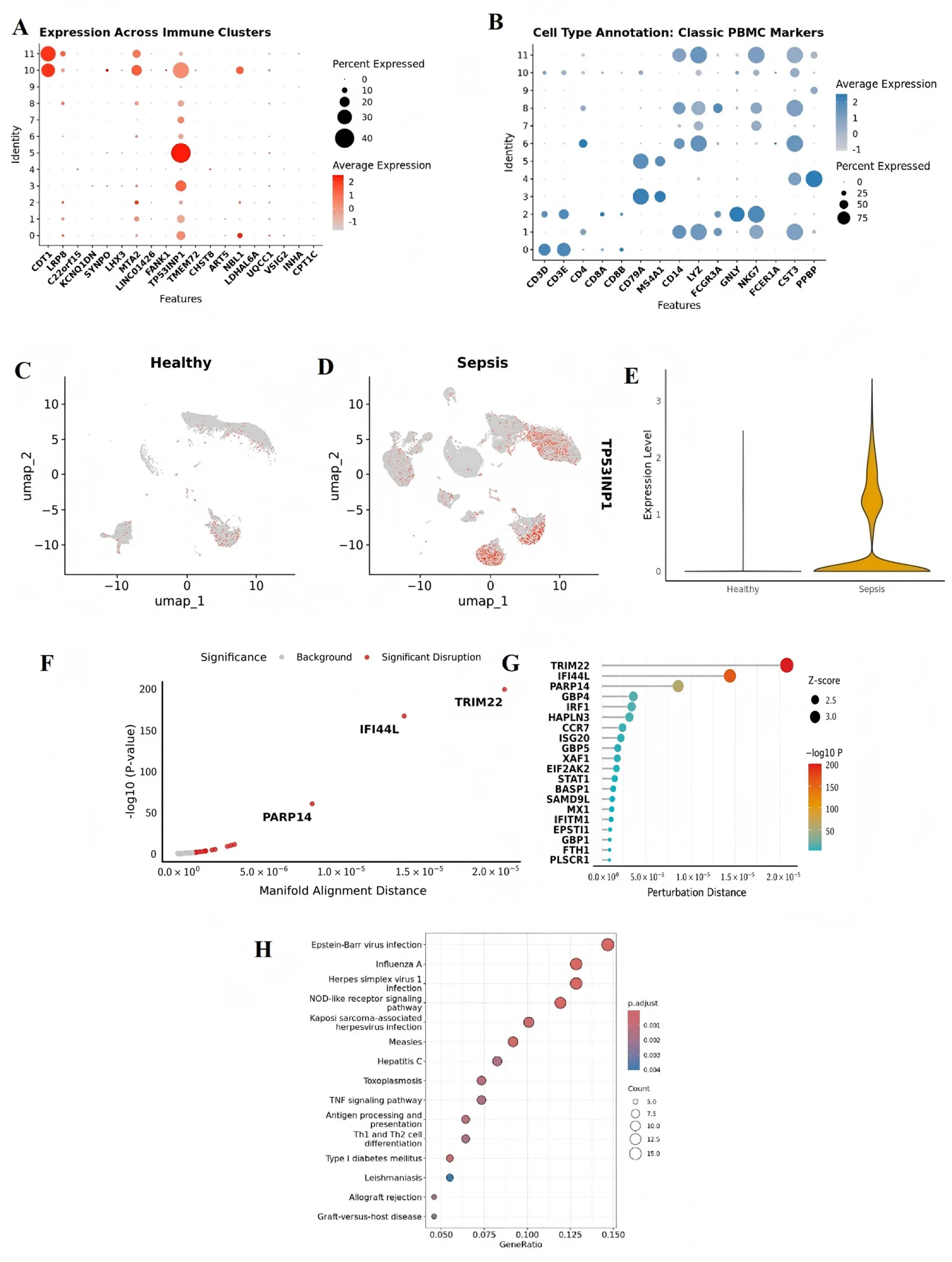
Cell-level TP53INP1 expression and computational network-perturbation analysis in the external GSE167363 sepsis dataset. (**A**,**B**) Candidate-gene and canonical marker expression across cell clusters; point size indicates percent expressed and color indicates average expression. (**C**,**D**) TP53INP1 expression in healthy and sepsis cells. (**E**) Cell-level TP53INP1 expression in B cells. (**F**,**G**) Predicted TP53INP1-associated network perturbations; nominal *p* values were used without multiple-testing correction. (**H**) KEGG enrichment of predicted perturbed genes; point size indicates gene count and color indicates adjusted *p* value.

The expression patterns of the candidate genes were examined across the annotated cell populations (Figure 5A). Cell identities were assigned on the basis of canonical lineage-marker expression (Figure 5B). TP53INP1 showed a prominent B-cell-enriched expression pattern in the external single-cell dataset. Feature and violin plots were further used to descriptively visualize TP53INP1 expression in B cells from sepsis and healthy-control samples (Figure 5C–E). Because these observations were not evaluated using a donor-aware statistical model, no inferential conclusion regarding differential TP53INP1 expression between the two groups was drawn.

### 3.6. Computational Network Perturbation Identifies TP53INP1-Associated B-Cell Gene Programs

Computational perturbation of TP53INP1 using scTenifoldKnk identified a set of genes with relatively large predicted network-perturbation distances (Figure 5F,G). Among the higher-ranking genes were several interferon-stimulated or antiviral-response genes, including TRIM22, IFI44L, and PARP14. Functional enrichment of the predicted perturbed gene set included pathways annotated as Epstein–Barr virus infection, influenza A, herpes simplex virus 1 infection, and NOD-like receptor signaling (Figure 5H). These pathway annotations reflect shared antiviral and inflammatory gene programs and should not be interpreted as evidence of coinfection or HAdV-7-specific regulatory effects. Overall, the analysis generates a computational hypothesis that TP53INP1 may be associated with B-cell antiviral-response networks in sepsis; it does not demonstrate that experimental alteration of TP53INP1 would produce these transcriptional changes.

## 4. Discussion

Severe HAdV-7 infection can be associated with critical illness and sepsis in children, but the host molecular alterations accompanying severe disease remain incompletely understood [7]. In the present study, we used an exploratory cross-cohort framework to examine these alterations at different levels of evidence. Whole-blood RRBS data from children with HAdV-7-associated sepsis provided the primary disease-specific methylation observations. An independent pediatric septic-shock transcriptomic dataset was subsequently used for candidate-gene prioritization, whereas an external sepsis single-cell dataset was used to examine the cellular expression context of the prioritized genes. Finally, computational network perturbation was applied to generate hypotheses regarding TP53INP1-associated gene programs. Because these molecular layers were obtained from different cohorts, the findings represent cross-cohort contextual evidence rather than direct multi-omic validation of an HAdV-7-specific mechanism. Because GSE26440 and GSE167363 differ from the present HAdV-7 cohort in disease etiology, patient characteristics, sample type, and profiling platform, the TP53INP1-associated signals identified across these datasets cannot be considered HAdV-7-specific. Instead, they may reflect broader molecular features of severe sepsis that warrant validation in disease-matched HAdV-7 cohorts.

The RRBS analysis identified group-associated whole-blood methylation differences, with 106 DMGs shared across the Survivor vs. Control, Non-survivor vs. Control, and Non-survivor vs. Survivor comparisons. These shared genes were used as a candidate-prioritization set and were not interpreted as a uniformly directional methylation signature. In addition, because methylation was measured in whole blood and leukocyte composition could not be formally adjusted, the observed differences cannot be attributed to specific immune-cell populations. Cross-cohort intersection with the GSE26440 turquoise module reduced the RRBS-derived candidate set to 19 genes. Importantly, although the turquoise module itself was not significantly associated with mortality, its overlap with the RRBS-derived DMGs provided an additional cross-cohort layer for candidate prioritization. The 19-gene overlap was therefore interpreted as an exploratory cross-cohort convergence that guided subsequent single-cell contextualization, rather than as a formal test of enrichment or mortality association.

Among the 19 candidate genes, TP53INP1 was selected for further exploration because it showed a prominent B-cell-enriched expression pattern in the external single-cell dataset. Alterations in B-cell number, phenotype, and function have been described in sepsis and septic shock [27,28,29], providing a biological context in which the observed expression pattern may be relevant. In the present analysis, TP53INP1 expression in sepsis and healthy-control B cells was visualized descriptively at the cell level. Because the single-cell dataset was derived from a clinically and etiologically distinct sepsis cohort and the comparison was not evaluated using a donor-aware statistical model, no donor-level differential-expression inference was made. Consequently, these observations do not establish that TP53INP1 is specifically dysregulated in B cells from children with HAdV-7-associated sepsis.

Mechanistically, TP53INP1 functions as a stress-responsive protein orchestrating p53-mediated cell cycle arrest and apoptosis [30]. Beyond classical apoptotic pathways, emerging evidence indicates that TP53INP1 is a regulator associated with oxidative stress responses and a modulator of autophagy under severe pathogenic burdens [31]. p53 also participates in interferon-mediated antiviral immunity by enhancing interferon signaling and the transcription of interferon-stimulated genes [32]. Pathway enrichment data suggested that sepsis-derived B cells were subjected to antiviral-response signaling and type I interferon signaling. In this hyper-inflammatory microenvironment, TP53INP1 is a stress-responsive regulator associated with p53-dependent cell-cycle control and apoptosis. In the external sepsis single-cell dataset, TP53INP1 expression was enriched in B-cell clusters. Network perturbation analysis predicted that alterations in TP53INP1 could affect regulatory programs involving TRIM22, IFI44L, PARP14, antigen presentation, TNF signaling, and antiviral responses. These results provide a testable hypothesis linking TP53INP1 to B-cell immune dysfunction; however, they do not demonstrate that TP53INP1 directly induces B-cell apoptosis or depletion in HAdV-7 sepsis. Experimental knockdown or overexpression studies are required to establish causality.

This study has several limitations. First, the RRBS discovery cohort was small, comprising 11 patients and 5 controls, which limited statistical power and the ability to adjust comprehensively for potential confounders. Although multiple-testing correction was applied, the small sample size may still increase the instability and false-positive risk of the methylation findings. Given the small discovery cohort, the stability and reproducibility of the identified methylation signals remain uncertain, and the 106 shared DMGs should be considered exploratory candidates requiring validation in larger independent cohorts. Second, whole-blood DNA methylation profiles are influenced by leukocyte composition, and complete leukocyte-subset data were unavailable; consequently, the observed methylation differences cannot be interpreted as cell-intrinsic epigenetic alterations. Because cell-composition correction could not be performed, we could not determine whether the identified methylation differences would persist after adjustment for leukocyte composition. Third, the bulk and single-cell transcriptomic datasets were derived from independent sepsis cohorts with clinical and etiological characteristics that differed from those of the pediatric HAdV-7 cohort, and no within-subject methylation–expression relationships were available. Fourth, the single-cell expression comparisons were conducted at the cell level and did not account for the non-independence of cells derived from the same donor, introducing a potential pseudoreplication bias. Fifth, the WGCNA-based candidate prioritization was exploratory, and TP53INP1 was selected for focused analysis based on its B-cell-enriched expression pattern rather than a prespecified composite ranking score. Finally, the scTenifoldKnk analysis was computational and did not constitute an experimental gene knockout; detailed information regarding cell subsampling, random seeds, and robustness across alternative network parameters was not retained in the archived analysis records. Because TP53INP1 was prioritized entirely through cross-cohort computational analyses, its expression, methylation status, and functional role were not independently validated in the present study. The proposed TP53INP1-associated B-cell hypothesis therefore requires validation in larger disease-matched cohorts using donor-aware transcriptomic analyses and direct functional experiments.

## 5. Conclusions

In summary, cross-cohort integration of whole-blood RRBS data from pediatric HAdV-7-associated sepsis with independent sepsis transcriptomic datasets prioritized TP53INP1 as a candidate gene with B-cell-enriched expression in an external single-cell dataset. Computational network perturbation further generated hypotheses linking TP53INP1 with antiviral and inflammatory B-cell gene programs. These findings do not establish an HAdV-7-specific TP53INP1 mechanism and should be regarded as hypothesis-generating observations requiring validation in larger disease-matched cohorts and functional experimental models.

## Figures and Tables

**Table 1:** Clinical characteristics and treatment information of children with HAdV-7-associated sepsis.

ID	Sex	Age (Months)	Admission Weight (kg)	Pre-Admission Illness Duration (Days)	Hospital Stay (Days)	PICU Length of Stay (Days)	Invasive Ventilation (Days)	HFV	ECMO	Outcome	PaO_2_/FiO_2_ Ratio	WBC Count (×10^9^/L)	Neutrophil Count (×10^9^/L)
1	Male	6	8	5	39	13	11	Y	Y	Survivor	93	13.3	7.76
2	Male	96	17	15	28	21	18	Y	Y	Survivor	43	5.6	4.07
3	Female	14	10	7	38	38	38	Y	Y	Survivor	60	6.2	5.1
4	Female	12	10	7	27	12	8	N	N	Survivor	97	2	1.29
5	Female	20	12	14	27	18	15	N	N	Survivor	94	1.6	0.67
6	Male	19	10	7	23	11	11	N	N	Survivor	138	2.7	1.48
7	Female	17	10	15	25	24	25	Y	N	Non-survivor	72	11.5	10.38
8	Male	7	10	10	7	7	7	N	Y	Non-survivor	36	7.1	4.5
9	Male	36	16	7	57	57	57	Y	Y	Non-survivor	66	2.7	1.35
10	Male	8	8	25	5	5	5	N	Y	Non-survivor	36	9.8	7.97
11	Male	10	9	7	24	22	22	Y	Y	Non-survivor	81	6.3	3

Abbreviations: PICU, pediatric intensive care unit; ECMO, extracorporeal membrane oxygenation; PaO_2_/FiO_2_ ratio, ratio of arterial oxygen partial pressure to the fraction of inspired oxygen; WBC, white blood cell; Neutrophil count, absolute neutrophil count. Y, yes; N, no. HFV, high-frequency ventilation.

**Table 2:** Clinical characteristics of patients with HAdV-7-associated sepsis stratified by survival status.

Characteristic	Survivors (n = 6)	Non-Survivors (n = 5)
Age, months	16.5 (12.5–19.8)	10.0 (8.0–17.0)
Male sex, n (%)	3 (50.0)	4 (80.0)
Admission weight, kg	10.0 (10.0–11.5)	10.0 (9.0–10.0)
Pre-admission illness duration, days	7.0 (7.0–12.3)	10.0 (7.0–15.0)
ARDS, n (%)	6 (100)	5 (100)
Invasive mechanical ventilation, n (%)	6 (100)	5 (100)
Invasive ventilation duration, days	13.0 (11.0–17.3)	22.0 (7.0–25.0)
HFV, n (%)	3 (50.0)	3 (60.0)
ECMO, n (%)	3 (50.0)	4 (80.0)
Vasoactive support, n (%)	6 (100)	5 (100)
PaO_2_/FiO_2_ ratio	93.5 (68.3–96.3)	66.0 (36.0–72.0)
WBC count, ×10^9^/L	4.15 (2.17–6.05)	7.10 (6.30–9.80)
Neutrophil count, ×10^9^/L	2.78 (1.34–4.84)	4.50 (3.00–7.97)
Sampling time	PICU admission	PICU admission

## Data Availability

The raw RRBS sequencing data are not publicly deposited because of ethical and patient-privacy restrictions associated with the original consent and study approval. De-identified derived data used for candidate prioritization, including the complete lists of 106 shared DMGs and 19 intersecting candidate genes, are provided as Supplementary Data S1 and S2. Additional de-identified analytical materials may be requested from the corresponding authors, subject to institutional and ethical requirements. The public transcriptomic datasets are available from GEO under accession numbers GSE26440 and GSE167363.

## Supplementary Materials

The following supporting information can be downloaded at: https://www.mdpi.com/article/10.3390/pathogens15090988/s1, Supplementary Data S1: Complete list of the 106 shared differentially methylated genes identified across the three pairwise comparisons; Supplementary Data S2: Complete list of the 19 candidate genes obtained by intersection of the RRBS-derived DMGs and the WGCNA turquoise module.

## Abbreviations

ARDS, acute respiratory distress syndrome; DMGs, differentially methylated genes; DMRs, differentially methylated regions; DNA, deoxyribonucleic acid; ECMO, extracorporeal membrane oxygenation; FDR, false discovery rate; GO, gene ontology; GO-BP, gene ontology biological processes; GS, gene significance; HAdV, human adenovirus; KEGG, Kyoto encyclopedia of genes and genomes; PaO_2_/FiO_2_ ratio, ratio of arterial oxygen partial pressure to the fraction of inspired oxygen; PBMCs, peripheral blood mononuclear cells; PCA, principal component analysis; PCR, polymerase chain reaction; PICU, pediatric intensive care unit; RRBS, reduced representation bisulfite sequencing; scRNA-seq, single-cell RNA sequencing; UMAP, uniform manifold approximation and projection; WGCNA, weighted gene co-expression network analysis.
