# Supplementary material for: Cross-Cohort Integration of Blood DNA Methylation and Sepsis Transcriptomes Prioritizes TP53INP1 as a Candidate Associated with B-Cell Transcriptomic Patterns in Pediatric HAdV-7-Associated Sepsis
Source: Pathogens. 2026 Sep 17;15(9):988. doi: 10.3390/pathogens15090988 (PMC13610302; doi:10.3390/pathogens15090988)
Supplement: Supplementary file 1 [file pathogens-15-00988-s001.zip › Supplementary Data S1.pdf]

Supplementary Data S1. Complete list of the 106 shared differentially methylated genes identified across the three pairwise comparisons.

Rank	Gene	Rank	Gene	Rank	Gene
1	TTLL10	36	CARHSP1	71	EDEM2
2	NADK	37	MIR9901	72	UQCC1
3	NBL1	38	LOC400553	73	HELZ2
4	CELA3A	39	MIR5189	74	LINC01426
5	LRP8	40	CDT1	75	TRPM2
6	RUSC1-AS1	41	APRT	76	SLC19A1
7	TRIM67	42	CBFA2T3	77	C22orf15
8	TMEM72	43	SPIRE2	78	EIF4E3
9	CTBP2	44	MYO1C	79	RPN1
10	FANK1	45	ALOX12	80	PRR23A
11	LRRC27	46	FGF11	81	RNF7
12	FUOM	47	MIR4521	82	C3orf80
13	LSP1	48	RPL26	83	NOP14-AS1
14	KCNQ1DN	49	MFSD6L	84	MFSD10
15	ART5	50	MIEN1	85	WDR19
16	LDHAL6A	51	ABI3	86	ACSL1
17	ZNF408	52	GNGT2	87	CMYA5
18	MTA2	53	LOC101928674	88	PCDHGA12
19	MIR7155	54	TSPAN10	89	SYNPO
20	LOC728975	55	REEP6	90	IRGM
21	ZDHHC24	56	ZNF57	91	NSD1
22	PTPRCAP	57	RETN	92	MIR3143
23	KCTD14	58	CEBPA	93	HIST1H2BK
24	VSIG2	59	CHST8	94	MEST
25	LOC101929384	60	ZNF571-AS1	95	MESTIT1
26	PYM1	61	LOC100631378	96	MIR8055
27	DGKA	62	EXOC3L2	97	NDUFAF6
28	LOC105369879	63	RTN2	98	TP53INP1
29	RPL6	64	TMEM160	99	EXOSC3
30	OASL	65	CPT1C	100	C9orf129
31	LINC02361	66	UCN	101	CCDC180
32	BEGAIN	67	KRTCAP3	102	SLC25A25
33	JAG2	68	INHA	103	CIZ1
34	CORO2B	69	ECEL1P2	104	LHX3
35	DDX11L10	70	MLLT10P1	105	DNLZ
				106	LINC01451
