# Supplementary material for: Cross-Cohort Integration of Blood DNA Methylation and Sepsis Transcriptomes Prioritizes TP53INP1 as a Candidate Associated with B-Cell Transcriptomic Patterns in Pediatric HAdV-7-Associated Sepsis
Source: Pathogens. 2026 Sep 17;15(9):988. doi: 10.3390/pathogens15090988 (PMC13610302; doi:10.3390/pathogens15090988)
Supplement: Supplementary file 1 [file pathogens-15-00988-s001.zip › Supplementary Data S2.pdf]

Supplementary Data S2. Complete list of the 19 candidate genes obtained by intersection of the RRBS-derived DMGs and the WGCNA turquoise module.

**Rank**	**Gene**	**Rank**	**Gene**
1	NBL1	11	MTA2
2	INHA	12	SYNPO
3	CDT1	13	LDHAL6A
4	UQCC1	14	FANK1
5	C22orf15	15	ART5
6	VSIG2	16	CHST8
7	CPT1C	17	KCNQ1DN
8	LINC01426	18	LHX3
9	TMEM72	19	LRP8
10	TP53INP1		
